# Parents’ expectations for the management of pediatric diarrhea in the clinical setting: perspectives of parents and physicians in Bangladesh

**DOI:** 10.1093/tropej/fmaf044

**Published:** 2025-11-11

**Authors:** Sarah A Dallas, Aparna Mangadu, Jyoti Bhushan Das, Olivia Hanson, Zahid Hasan Khan, Mohammad Ashraful Amin, Ishtiakul Islam Khan, Md Taufiqul Islam, Debashish Biswas, Mohammad Saeed Munim, Ridwan Mustafa Shihab, Ahmed Nawsher Alam, Tahmina Shirin, Firdausi Qadri, Jane Putnam, Eric J Nelson, Daniel T Leung, Ashraful Islam Khan, Melissa H Watt

**Affiliations:** Honors College, University of Utah, Salt Lake City, UT, 84113, United States; Division of Infectious Diseases, Department of Internal Medicine, Spencer Fox Eccles School of Medicine at the University of Utah, Salt Lake City, UT, 84132, United States; Infectious Diseases Division, International Center for Diarrhoeal Disease Research, Bangladesh (icddr, b), Dhaka, 1212, Bangladesh; Spencer Fox Eccles School of Medicine at the University of Utah, Salt Lake City, UT, 84132, United States; Infectious Diseases Division, International Center for Diarrhoeal Disease Research, Bangladesh (icddr, b), Dhaka, 1212, Bangladesh; Infectious Diseases Division, International Center for Diarrhoeal Disease Research, Bangladesh (icddr, b), Dhaka, 1212, Bangladesh; Infectious Diseases Division, International Center for Diarrhoeal Disease Research, Bangladesh (icddr, b), Dhaka, 1212, Bangladesh; Infectious Diseases Division, International Center for Diarrhoeal Disease Research, Bangladesh (icddr, b), Dhaka, 1212, Bangladesh; Health System and Population Studies Division, International Center for Diarrhoeal Disease Research, Bangladesh (icddr, b), Dhaka, 1212, Bangladesh; School of Population and Global Health, The University of Western Australia, Perth, WA, 6009, Australia; Health System and Population Studies Division, International Center for Diarrhoeal Disease Research, Bangladesh (icddr, b), Dhaka, 1212, Bangladesh; Health System and Population Studies Division, International Center for Diarrhoeal Disease Research, Bangladesh (icddr, b), Dhaka, 1212, Bangladesh; Institute for Epidemiology, Disease Control and Research (IEDCR), Dhaka, 1212, Bangladesh; Institute for Epidemiology, Disease Control and Research (IEDCR), Dhaka, 1212, Bangladesh; Infectious Diseases Division, International Center for Diarrhoeal Disease Research, Bangladesh (icddr, b), Dhaka, 1212, Bangladesh; Honors College, University of Utah, Salt Lake City, UT, 84113, United States; Department of Pediatrics, University of Florida, Gainesville, FL, 32209, United States; Division of Infectious Diseases, Department of Internal Medicine, Spencer Fox Eccles School of Medicine at the University of Utah, Salt Lake City, UT, 84132, United States; Infectious Diseases Division, International Center for Diarrhoeal Disease Research, Bangladesh (icddr, b), Dhaka, 1212, Bangladesh; Department of Population Health Sciences, Spencer Fox Eccles School of Medicine at the University of Utah, Salt Lake City, UT, 84108, United States

## Abstract

Diarrheal diseases are a leading cause of morbidity and mortality in children globally, and antibiotics are often inappropriately used in the management of pediatric diarrhea. This study explores how parents’ expectations influence the prescription of antibiotics for pediatric diarrhea in Bangladesh. We used qualitative methods to explore parents’ expectations when bringing their child with diarrhea to hospital and how physicians perceive and manage expectations. We conducted interviews with 36 parents and 18 hospital physicians across three hospitals. Data analysis followed an applied thematic analysis framework. Parents expected a higher quality of care in the hospital setting, including diagnostic testing, medication, and psychosocial support. Most parents did not expect antibiotics as treatment for pediatric diarrhea, yet most parents expressed a belief that antibiotics were superior to other medications. Physicians recognized this parental belief about antibiotic superiority, but some mistakenly assumed parents universally expected antibiotics. Physicians stated that the most common form of managing parents’ expectations is via educational counseling. Physicians’ assumptions that parents expect to receive antibiotics may lead to inappropriate antibiotic prescription and be a source of frustration for both parties. Shared decision-making interventions can assist physicians in exploring and managing parent expectations to promote antibiotic stewardship.

## Background

Diarrheal diseases are the third leading cause of morbidity and mortality in children globally [1] and are especially prevalent in low- and middle-income countries (LMICs), where they greatly impact children under five [2, 3]. Most pediatric diarrhea cases in LMICs are acute, of infectious origin, and self-limiting. Due to this, etiologic determination is often unnecessary for diarrheal cases, which can be treated symptomatically with rehydration therapy [4]. In the majority of cases, in the absence of severe symptoms, pediatric diarrhea can be treated in an outpatient setting without antibiotics [5].

Antibiotics are often overused in low resource settings to treat pediatric diarrhea, even when clinically unnecessary [6]. This inappropriate antibiotic use can result in adverse side effects, unnecessary economic burden on the patient and system, and antibiotic resistance [7]. In Bangladesh, diarrhea is one of the leading causes of antibiotic use [8], driven by multiple factors including lack of regulation and access to diagnostic tools, and the expectations of patient’s families and physicians [9, 10].

In recent years, pathogens that cause many common illnesses have developed resistance to common antibiotics [11, 12]. While the inappropriate use of antibiotics and antibiotic resistance are global issues, they’re especially pronounced in resource-poor settings like Bangladesh, where excess antibiotic prescribing practices and patient non-compliance with antibiotic treatment regimens significantly contribute to antimicrobial resistance (AMR) [13].

While healthcare providers’ clinical decisions for treating pediatric diarrhea are largely informed by factors such as patient symptoms and disease etiology, parental expectations have been observed to influence treatment [14]. To maintain patient satisfaction, healthcare providers may feel pressured to alter their treatment plans and, in some instances, prescribe antibiotics even when clinically unnecessary [15].

To understand how parental expectations, influence the clinical management of pediatric diarrhea in Bangladesh, this study used qualitative methods to explore the expectations of parents of children with diarrhea and how physicians perceive and manage these expectations.

## Methods

### Overview

This exploratory qualitative study in Bangladesh examined perspectives from two groups: (i) parents of children seeking care for diarrhea and (ii) physicians treating pediatric diarrhea at government hospitals.

### Study sites and participants

**Figure 1.**
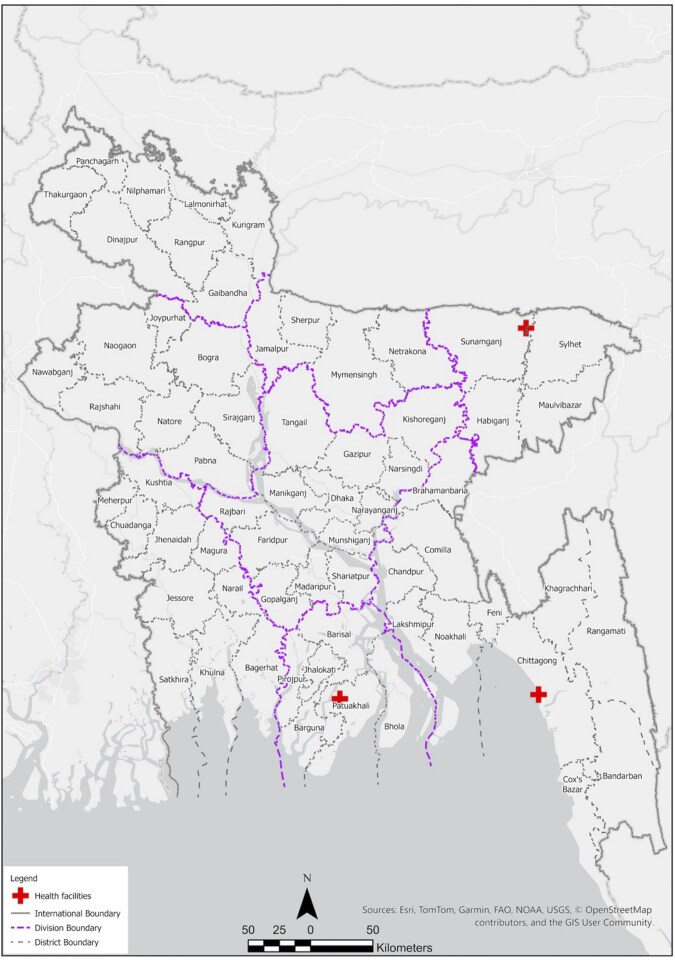
Locations of clinical sites.

Participants were recruited from three government healthcare facilities across three districts of Bangladesh (Fig. 1). Facility 1 was an urban specialized referral hospital, Facility 2 was an urban district hospital, and Facility 3 was a rural sub-district hospital. The facilities serve a high volume of patients, from 50 000 to 200 000 patients per year.

On selected clinic days, the research team approached families of children under 5 years whose primary complaint was diarrhea and enrolled 36 parents (12 per site). In close collaboration with hospital leadership, 18 healthcare providers (6 per site) were enrolled. We continued recruitment until no new themes or insights emerged from additional interviews, indicating that data saturation had been achieved.

### Procedures

Data were collected from June to August 2023, with interviews conducted in private settings in each hospital. Interviews followed a semi-structured guide with open-ended questions and follow-up probes that were developed based on the theory of planned behavior (TPB) [16].

The interview guide for parents (Supplementary Appendix S1) covered expectations regarding: (i) treatment quality, (ii) diagnostic testing, (iii) antibiotic usage, and (iv) other management practices when visiting the hospital for pediatric diarrhea. Parent interviews lasted on average 31 min. The clinician interview guide (Supplementary Appendix S2) covered physician perspectives of parents’ expectations and how expectations influence clinical practice. Physician interviews lasted on average 51 min. Interviews were audio recorded, transcribed verbatim in Bangla, and translated to English for analysis.

### Qualitative data analysis

Analysis followed an applied thematic analysis framework [17] in the following phases: familiarization with the transcripts, identification of major themes, development of a codebook, coding, synthesis of analytic memos, interpretation, and analysis. Coding was done in NVivo (version 14 pro) [18]. The codebook was developed to identify emerging themes in the following domains: (i) parents’ expectations of care and (ii) physician’s interpretation and management of parents’ expectations.

### Ethics

The study was approved by the Ethical Committee of the International Centre for Diarrheal Disease Research, Bangladesh (icddr, b) #22114 and the University of Utah Institutional Review Board. Prior to interviews, the team explained the study and obtained written informed consent.

## Results

### Description of the sample

The parent interviews (*n* = 36) included 25 mothers and 11 fathers with an average age of 27 years (range: 18-43 years). The pediatric patients were an average of 16.25 months in age (range: 5 months–3 years). Clinician participants (*n* = 18) had an average of 10.33 years of experience (range: 0.25–26 years) and were mostly male (13/18).

### Parents’ expectations of care

**Figure 2.**
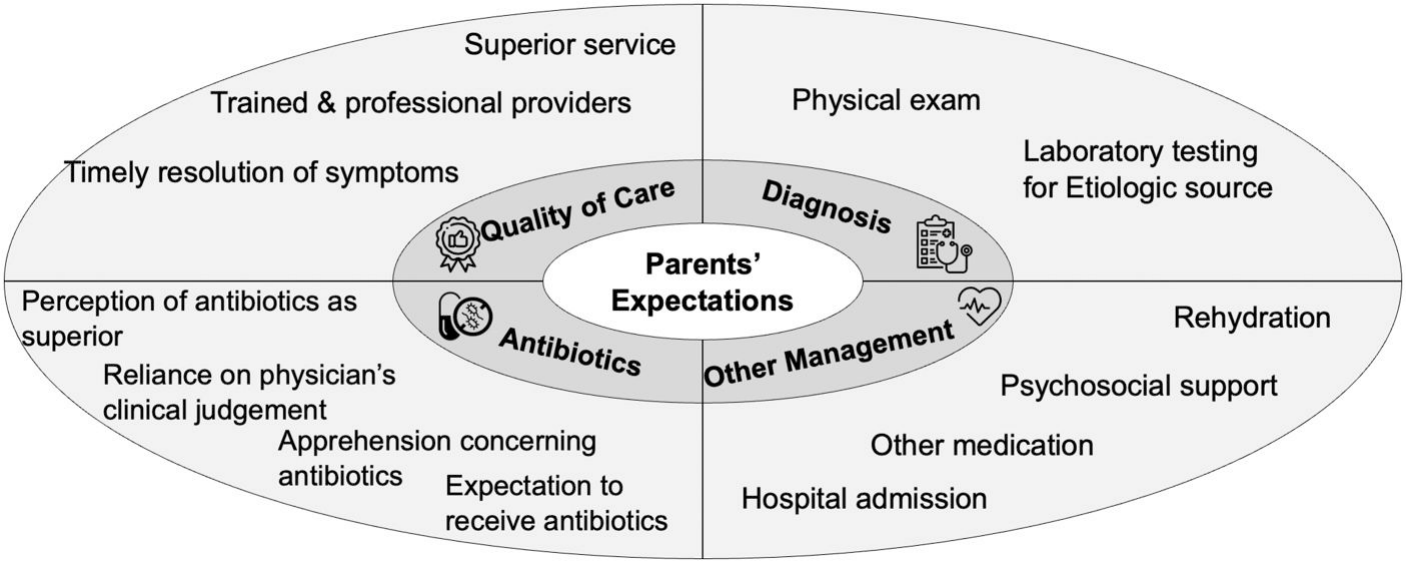
Summary of qualitative findings for parents’ expectations of care for pediatric diarrhea.

Interviews with parents identified expectations concerning the treatment of their child’s diarrhea around four domains: (i) quality of care, (ii) diagnostic testing, (iii) antibiotic usage, and (iv) other management practices. The emerging themes in each topic are summarized in Fig. 2.

#### Quality of care

Several parents initially sought medical care for their children outside of government hospitals, at smaller or informal facilities but were unsatisfied with the treatment received. Given this, parents discussed the expectation of receiving more effective treatment in the hospital setting.

“I came here because I went to several doctors and gave my child those medicines, but his condition wasn’t improving at all. That’s why I came. I thought that if I could get treatment here, then his condition would be okay.” (Mother of 8-month-old taken to Facility 2)

Parents expected timely symptom resolution and were disappointed when treatment did not meet this expectation. Several parents associated treatment quality with duration of recovery.

“We had a demand that my child would get well soon. We can return to our home as soon as possible. Doctors from this hospital provide good treatment.” (Mother of 5-month-old taken to Facility 2)

Participants expected providers to be competent and have a professional disposition. Several participants indicated that provider communication was a major factor in their satisfaction.

“The young doctors who sit in the emergency room are smart and knowledgeable. The treatments that they give are actually good. If I talk to them, they respond well and give several solutions.” (Father of 17-month-old taken to Facility 3)

#### Diagnostic testing

Several participants expected a physical exam during the visit and expressed disapproval when one was not performed, citing the concern of an uninformed diagnosis.

“A doctor came to see my child, but his temperature was 104 degrees and the doctor didn’t even touch him to understand the severity of my child’s fever.” (Mother of 45-month-old taken to Facility 2)

Over half of participants expected diagnostic testing during the visit, viewing them as necessary for understanding the cause of their child’s diarrhea and ensuring evidence-based treatment.

“When I visited Dr X, he did not give me any medical tests. Without having any medical tests, he prescribed iron and vitamins. Dr Y also gave me those medicine, but with medical tests. That is why I am satisfied with Dr Y’s treatment.” (Mother of 13-month-old taken to Facility 2)

Conversely, a few participants felt that testing was only important if recommended by the doctor and ultimately put their trust in the doctor’s experience and best judgment.

“If the doctor suggests doing the test, I will do the test. I must follow the doctor’s instruction.” (Mother of 31-month-old taken to Facility 3)

The cost of diagnostic testing was a concern for many parents, one noting that it is important for physicians to examine the patient before ordering diagnostics to avoid unnecessary, expensive tests.

“Sometimes doctors order too many tests which is not really necessary. Moreover, most of the people are not well-off to do all of those tests. If the doctors examine the patients and suggests only the tests which are actually necessary, it would be beneficial.” (Mother of 16-month-old taken to Facility 1)

#### Antibiotic usage

Participants generally had a favorable attitude toward antibiotics, stating, for example, that antibiotics “work faster, and the child becomes well in a short period of time” *(Mother of a 19-month-old taken to Facility 2)*. That said, the majority of participants expressed no expectation for their child to receive antibiotics as part of the initial treatment.

“It’s better not to give a child antibiotics if their diarrhea is reduced.” (Mother of 11-month-old taken to Facility 2)

Many parents expressed concern about the side effects of antibiotics and possible detriments to their children’s long-term health.

“We know antibiotics are detrimental to health, so it is better not to give antibiotics to children.” (Mother of 10-month-old taken to Facility 3)

The majority of parents, even those that did not expect antibiotics as an initial part of treatment, stipulated that they would expect antibiotics to be given under certain circumstances. In general, antibiotics were expected with increased severity or duration of symptoms, if new symptoms appeared, or if other medications did not improve symptoms.

“I think it is better not using antibiotic at the initial level treatment of the pediatric diarrhea patient.… He may face some problem if he takes antibiotics randomly. So, at first, he needs to take normal medicines. If he doesn’t get well with those medicines then he may give antibiotics with the suggestion of the doctor.” (Father of 7-month-old taken to Facility 2)

Several participants stated that they did not know much about antibiotics, their properties, or when they should be prescribed. These parents noted a reliance on the physician’s ability to diagnose and treat pediatric diarrhea patients.

“I don’t know [about antibiotics]. Doctors know. I think they can say better.” (Mother of 13-month-old taken to Facility 3)

About a third of participants felt that antibiotics are necessary to effectively treat pediatric diarrhea and would not feel satisfied with their care if they did not receive antibiotics.

“These days, nobody recovers from any illness without antibiotics. Normal medicines do not work properly. So, antibiotics are necessary for all kinds of disease to make a fast recovery. Therefore, I think if a child has had diarrhea for several days, he should be given antibiotics.” (Mother of 29-month-old taken to Facility 1)

#### Other management practices

Other than diagnostic tests and antibiotics, participants also stated that it was important that the physician provided reassurance about the child’s condition. Many parents stated that receiving reassurance and guidance improved the quality of care.

“I expect that the doctors will give me hope about the health of my child.” (Mother of 13-month-old taken to Facility 2)

Many participants also spoke about the importance of receiving medications other than antibiotics for the management of their child’s diarrhea. Several parents held an expectation that medicine provided by the hospital would be of high quality and more likely to cure illness than medicine provided by outside doctors or pharmacies.

“I thought that medicine provided by the hospital is better than the outsides… Here the medicines are good, the doctors treat you well, and the diagnosis is also better than the others. They treat the patient carefully. That influenced us to take the baby to the hospital.” (Mother of 13-month-old taken to Facility 3)

A few participants mentioned an expectation to receive rehydration therapy.

“As we know, there are facilities to provide saline when patients are admitted to the hospital. For any patient with diarrhea, saline is essential.” (Father of 10-month-old taken to Facility 1)

### Physicians’ perceptions of parents’ expectations

Interviews with physicians described how they interpret parents’ expectations and how they manage these expectations. Physicians felt that the primary expectation of parents was that their child would experience a rapid recovery. For physicians, this created a tension when rapid recovery was not possible.

“Mothers want immediate relief from the symptoms of the [child’s] disease. No matter how big a doctor you are, the child’s mother will want to take her to another doctor if she is not recovering. … As much as we say to a mother or guardian, it takes time to heal, you can’t force her to stay.” (MD, 16 years of practice)

Physicians noted that there are parents who request diagnostics and those who do not. While request for diagnostics was mixed, tension generally arose when parent’s and physician’s ideas about diagnostics did not align.

“We have mixed types of patients admitted here. Suppose I like to do the test, but the patient’s financial condition is poor. In the other case, I like to do the test, and the patient’s financial condition is stable, but they do not want to do the test.” (MBBS, 1.5 years of practice)

Physicians observed that parents who brought their child to the hospital expected more advanced medical interventions than what might have been available at lower-level facilities. This generally included medication (specifically antibiotics) and IV fluids. Physicians perceived that access to antibiotics, ideally free of charge, is a driving motivation for parents to bring their child to the hospital.

“(Antibiotic demand) is a common issue. Not only for diarrhea, but for every disease, they insist on getting antibiotics, saying, ‘Have they come to the hospital just for saline?’ It has become a common scenario. … 80% of them will complain about only getting saline.” (MBBS, MPH, 23 years of practice)

### Physicians’ management of parents’ expectations

Physicians stated that their main tools for managing parents’ expectations were counseling and education. This often includes educating parents on typical presentation and duration of pediatric diarrhea and reassuring them of the efficacy of symptomatic treatment.

“In this situation, I give them counseling. I tell them we don’t give treatment for diarrhea; rather, we give treatment for dehydration and the fluids that are going away from the patient’s body. I tell them to give the patient foods such as green bananas and [poha], which are good at stopping pooping. As it is a belly problem, we have nothing to do here; it will be solved automatically if you give it time. It may take several days, but don’t panic.” (MBBS, 1.5 years of practice)

Some physicians mentioned that they counsel against diagnostic testing, even if the parents request it: “I do not advise investigation, even if the patient wants it, unless it is very necessary.” *(Medical doctor, 16 years of practice)*. Other physicians mentioned that they will conduct testing to satisfy parents’ expectations even when it is not clinically necessary.

“In private chambers or where I do my private practice, patients are connected to me and there we have to advise diagnostic tests for the patient’s satisfaction. In that case, stewardship may not be possible to maintain. Along with necessary tests, some unnecessary tests also have to be given due to the patient’s demand.” (MBBS, 4 years of practice)

One physician stated that they initially deny parental requests for antibiotics and attempt to educate them but are willing to prescribe antibiotics if parents persist.

“We explain to them that if this patient takes antibiotics that are not actually necessary, then it will reduce his immunity. In the future, you won’t be able to use any normal antibiotics; you have to use the stronger one…. To some extent, they [parent’s expectations] do [influence prescription practices]. If they think antibiotics should be prescribed, we prescribe antibiotics.” (Diploma in Child Health, 22 years of practice)

While most physicians agreed that antibiotics are usually not necessary for pediatric diarrhea, they emphasized the importance of family satisfaction. As a compromise, they often discharge families with instructions for rehydration and symptom management and information about antibiotics should their symptoms persist.

“It [parent expectations] affects negatively. In my practice, I write the normal treatment and then write the name of the antibiotics in small size and tell them to start it if not improved. That is how the patient’s caregivers also get satisfied.” (MBBS, 4 years of practice)

## Discussion

Antibiotic resistance, driven by inappropriate antibiotic use, is a growing global crisis [12], especially in resource-poor settings like Bangladesh, where antibiotic usage for the treatment of pediatric diarrhea is a significant contributor [9, 10]. While antibiotics are not necessary in the treatment of most pediatric diarrhea cases due to their self-limiting nature, factors like parents’ expectations can influence physicians’ prescribing habits. In this study, we aimed to understand the expectations that parents hold when visiting the hospital for pediatric diarrhea treatment. This knowledge will help to improve communication between parents and clinicians and sustain antibiotic stewardship strategies.

Across clinical settings, antibiotic prescriptions, including those for pediatric diarrhea, are often driven by parents’ expectations and demand [19, 20]. Parental influence on clinician antibiotic prescribing practices has previously been documented in Bangladesh [21]. In our study, over half of parents did not expect antibiotics as a primary treatment for their child’s diarrhea and expressed concerns about antibiotic use in children, despite viewing them as superior medications overall. Parents primarily wanted timely symptom resolution and deferred to the medical team for the best care. Physicians often misinterpreted this desire for quick recovery as demand for antibiotics, leading to unnecessary antibiotic prescriptions.

This physician–parent communication gap suggests that the physicians may sometimes misinterpret parental expectations. Similar miscommunications have been found in relation to antibiotic prescription practices for upper respiratory tract infections [22]. Remedying this gap requires clear communication and shared decision-making practices [23], where providers and patients discuss evidence, treatment options, risks, benefits, and uncertainties. Given that parents often seek care with the expectation and hope of a prompt recovery for their child, effectively communicating prognosis with clinical empathy and appropriate reassurance not only supports judicious antibiotic use but also reinforces trust in the healthcare system and encourages future care-seeking behavior.

The study has limitations that should be considered. The study was conducted in three clinical facilities in Bangladesh, which limits its generalizability. Additionally, this study is limited by its qualitative design, which restrict the generalizability of our findings. Further research using larger, representative samples and quantitative methods is needed to better understand possible avenues of miscommunication between clinicians and caregivers and how they may contribute to inappropriate antibiotic use. The validity of the findings may be affected by social desirability bias, as participants could have tailored their responses to align with perceived expectations of the researchers or to present themselves in a favorable light. While the findings provide insights on drivers of antibiotic resistance, the role of antibiotic use in agriculture, livestock, and veterinary settings [24] should also be considered in comprehensive antibiotic stewardship efforts.

In conclusion, our findings highlight the importance of addressing communication gaps between parents and physicians to promote antibiotic stewardship in pediatric diarrhea treatment. Misaligned perceptions, particularly the assumption that parents equate prompt recovery with antibiotics, may contribute to unnecessary antibiotic use. Implementing evidence-based guidance, shared decision-making practices, and clear, empathetic communication can help bridge this gap, reducing inappropriate antibiotic use, combating antibiotic resistance, and promoting patient trust and satisfaction.

## Supplementary Material

fmaf044_Supplementary_Data
