# Supplementary material for: Parents’ expectations for the management of pediatric diarrhea in the clinical setting: perspectives of parents and physicians in Bangladesh
Source: J Trop Pediatr. 2025 Nov 11;71(6):fmaf044. doi: 10.1093/tropej/fmaf044 (PMC12604749; doi:10.1093/tropej/fmaf044)
Supplement: fmaf044_Supplementary_Data [file fmaf044_supplementary_data.zip › Appendix 1.pdf]

Interview Guide for Patient Caregivers

The purpose of our interview today is to get your feedback as a caregiver of a child who is presenting to health care with a primary complaint of diarrhea. We are mainly interested in understanding the expectations you have in getting the best care for your child.

As a reminder, your conversation with me today is for research purposes only. Nothing you say today will be shared with your doctor or any member of your clinical team. We hope you will feel comfortable talking openly and honestly about your experiences as a parent or caregiver. If any of my questions are not clear, please let me know and I can repeat or reword the question.

Do you have any questions before we begin?

I. Introduction: experience with childhood diarrhea

First, I'd like to talk to you about the events leading up to coming to this clinic/hospital for your child's diarrhea.

1. Please tell me briefly why you decided to come to the clinic/hospital for your child's diarrhea.
    a. What were your main worries or concerns about your child's condition?
2. Prior to coming to this clinic/hospital, did you seek care anywhere else? (probe: village doctor, pharmacy, traditional healer)
3. Prior to coming to this clinic/hospital, did you use any medications to help with your child's diarrhea? (If yes, describe)

II. Attitudes

Now I want to talk about what you expect when you bring your child with diarrhea to the clinic/hospital, and what is important to you.

1. When presenting to this clinic/hospital, what were you expecting to receive from the visit?
    a. In what ways did the visit meet your expectations?
    b. In what ways did the visit *not* meet your expectations?
2. For some families, it's very important for them to know exactly what is causing their child's diarrhea. How important is it for you to know this?
    a. Why do you say that this is important / not important?
    b. Do you need to have a test to determine the cause, or is it enough for the doctor to tell you what they think is causing the diarrhea?
3. For some families, it's very important for them to get medication (like antibiotic pills) for their child's diarrhea. How important is it for you to get treatment when your child has diarrhea?
    a. Why do you say that this is important / not important?
    b. [If important] What do you think that antibiotic pills do for your child?

III. Subjective norms

Thank you for telling me about your own expectations for how you want your child with diarrhea treated. Now I'd like to discuss how your friends and family think children with diarrhea should be treated.

1. In general, how do people in your community take care of a child when they have diarrhea?

    a. What signs and symptoms are most worrying / concerning?
    b. How do you decide what kind of care the child needs?
    c. How do you decide whether to give a child oral rehydration at home?
    d. How do you decide whether to withhold food / drink due to diarrhea?
2. How do you decide whether to take the child to the clinic?
    a. How do you decide where to seek care?
    b. Are there any times you might want to bring your child to the clinic, but don't?
    c. What are the barriers of taking the child to the clinic?
3. Who in your household makes the decision about how to treat a child with diarrhea, and when it is important to take the child to the clinic?

IV. Self-efficacy

Next I'd like to talk about how you talk with the nurses and doctors about your child's care when you come here.

1. Some people find it difficult to talk to providers about the care they would like to see for their child. How comfortable do you feel talking with your doctor about the treatment you would like for your child's diarrhea?
    a. What would make you more comfortable to talk to your doctor about your child's treatment?
    b. Who else in the family talks to the doctors and nurses about the sick child? What different roles do people in the family have in talking to the doctors and nurses?
2. How do you and other members of your family want to be involved in decisions about care for your child's diarrhea?
    a. What does being involved look like for you?

V. Behavioral intentions

Now I'd like to talk to you about the use of antibioitics for children with diarrhea.

1. In what circumstances do you think antibiotics are necessary when a child has diarrhea?
2. Where do people in your community get antibiotics? [NOTE: probe for a complete list]

There is a lot of discussion these days about over-use or inappropriate use of antibiotics. By antibiotics, I mean drugs like metronidazole, ciprofloxacin, azithromycin. The reason this is important is because if people use too many antibiotics, or don't use them correctly, then they can cause side effects, and in the long term, they can stop working.

3. Have you ever heard about this issue before? Tell me what you've heard.
4. As a caregiver for a sick child, what do you see as your personal role to reduce antibiotic use?
5. What drawbacks, if any, do you see of using antibiotics that are not clinically necessary?

VI. Utility and feasibility of eCDSTs

Our research team is in the process of developing a tool that providers would use to care for children with diarrhea. The tool would help them to make decisions about whether or not to order a diagnostic test, and whether or not to prescribe antibiotics.

1. How would you feel about your doctor or clinical using an application on their phone or computer to make a decision about whether or not they order a laboratory test to understand the origins of a case of pediatric diarrhea?
    a. Would this make you feel better or worse about the care your child receives? Why?

2. How would you feel about your doctor or clinical using an application on their phone or computer to make a decision about whether or not they prescribe an antibiotic for a case of pediatric diarrhea?
    a. Would this make you feel better or worse about the care your child receives? Why?
3. What information would you like the provider to share with you when using this tool?

I have reached this end of my questions. Do you have anything to add on this topic?
