# Supplementary material for: Parents’ expectations for the management of pediatric diarrhea in the clinical setting: perspectives of parents and physicians in Bangladesh
Source: J Trop Pediatr. 2025 Nov 11;71(6):fmaf044. doi: 10.1093/tropej/fmaf044 (PMC12604749; doi:10.1093/tropej/fmaf044)
Supplement: fmaf044_Supplementary_Data [file fmaf044_supplementary_data.zip › Appendix 2.pdf]

Interview Guide for Clinicians

The purpose of our interview today is two-fold. First, we want to understand how health care providers like you manage complaints of pediatric diarrhea. We are interested in both your use of diagnostic labs, and your use of treatments like antibiotics. Second, we want to get your feedback on the potential utility of a clinical decision support tool to help providers optimally manage cases of pediatric diarrhea.

As covered in the consent form, our conversation today will be used for research purposes only. When we present the results, we will aggregate everything we hear so that no individual is identified. We hope you will feel comfortable talking openly and honestly about your experiences as a clinician. If any of my questions are not clear, please let me know and I can repeat or reword the question.

Do you have any questions before we begin?

I. Introduction: experience taking care of children with diarrhea

First, I'd like to hear about your role as a clinician in this clinic/hospital, and your experience taking care of young children (under 5 years of age) who present with a complaint of diarrhea.

1. Your clinical schedule may vary, but please estimate for an average month, how often do you treat children under 5 who have a complaint of diarrhea?
    a. In what setting do you care for these children? (e.g., out-patient, in-patient)
    b. How does the presentation and frequency vary by season?

2. How would you describe the background of the young children who are brought here with diarrhea?
    a. Where do they come from geographically? (urban or rural, distance from the clinic/hospital)
    b. What is their economic situation?

3. During the summer/monsoon season, what does a typical case of diarrhea looks like in children who are brought to this hospital?
    a. Signs, symptoms, main complaints
    b. Dehydration level (none, some severe)
    c. Prior treatment before arriving at this clinic/hospital (from whom/where, any antibiotic use)
4. How are the cases of diarrhea different in other seasons?
    a. Signs, symptoms, main complaints
    b. Dehydration level (none, some severe)
    c. Prior treatment before arriving at this clinic/hospital (from whom/where, any antibiotic use)

5. Let's consider a previously health 2-year-old with 36 hours of diarrhea. How would you treat this case? (probe: diagnostics, treatment)

    a. What features, if present, might change your approach?

II. Behavioral intention

Now I'd like to talk to you about how you make clinical decisions when you see a child with diarrhea. We are especially interested in decisions about diagnostics and decisions about antibiotic use.

First, let's talk about diagnostic work up. By this we mean doing a stool-based diagnostic test to help the management of a case of diarrhea.

1. What diagnostic tests are available at your practice setting for managing children with diarrhea?
    a. How often do you use a diagnostic test on a child with diarrhea?
    b. Which diagnostic tests do you use?
    c. Is there a diagnostic test you wish you had available?

2. [IF STOOL BASED DIAGNOSTIC TESTS ARE AVAILABLE/USED] How do you decide whether or not to order a stool-based diagnostic test for a child with diarrhea?
    a. What pros and cons do you consider when deciding whether or not order a stool-based diagnostic test?
    b. How does the cost of diagnostic tests influence your decision making? (Does it vary by patient?)
    c. In which circumstances are you more likely to order a stool-based diagnostic test?
    d. What are your expected / desired outcomes from a diagnostic test?
    e. In which circumstances are you less likely to order a stool-based diagnostic test? Why?

3. Now, let's talk about use of antibiotics in the management of a child with diarrhea.
    a. How often do you give or recommend antibiotics for a child with diarrhea?
    b. Which antibiotic(s) do you prescribe for diarrhea?
    c. What factors determine your selection of which antibiotic you prescribe?

4. How do you decide whether or not to prescribe an antibiotic for a child with diarrhea?
    a. What pros and cons do you consider when deciding whether or not to prescribe an antibiotic for a child with diarrhea?
    b. How does the cost of antibiotics influence your decision making? (Does it vary by patient?)
    c. In which circumstances are you more likely to prescribe antibiotics?
    d. What types of antibiotics would you consider prescribing?
    e. What are your expected / desired outcomes?

III. Subjective norms (patients/caregivers)

Now I'd like to ask you about your conversations with the parents or caregivers of your patients, and how this informs your decisions about order a stool-based diagnostic test or giving an antibiotic.

1. What do you think are most families' expectations when they present with a child with diarrhea?
    a. In your opinion, how important is a diagnostic test for the family?
    b. In your opinion, how important is an antibiotic prescription for the family?

2. How do you communicate with families about their expectations?
3. How do families’ expectations inform your practice?

IV. Self-efficacy

As a provider, it can be difficult to know whether or not you are making the best decisions for your patient. You have told me about the information you use to make decisions about ordering a diagnostic test or giving antibiotics to a patient. Now I’d like to know how confident you feel about these decisions, and what additional information you would like to make the decision.

1. For a case of pediatric diarrhea, what information do you rely upon to make a decision about ordering a stool-based diagnostic lab? (probe: information about the patient, information about the broader environment)
    a. How confident do you feel about your ability to make the best decision of whether or not to order a diagnostic lab?
    b. What information would make you feel more confident?

2. What information do you rely upon to make a decision about prescribing an antibiotic for a child with diarrhea? (probe: information about the patient, information about the broader environment)
    a. How confident do you feel about your ability to make the best decision of whether or not to prescribe an antibiotic?
    b. What information would make you feel more confident?

V. Attitudes and Social Norms (clinical stakeholders)

Now, I’d like to talk to you about something called “diagnostic stewardship”.

1. When you hear the term “diagnostic stewardship”, what does this mean to you? [If term is not known, define: “Diagnostic stewardship” means that diagnostics are limited resources, and clinicians need to be careful about when and how they use these resources.]
    a. How does this inform your clinical practice?
    b. Is there a culture of diagnostic stewardship at your clinical practice location? Please describe.
    c. What are the main challenges that clinicians like you face in implementing diagnostic stewardship?

2. When you hear the term “antimicrobial stewardship”, what does this mean to you? [If term is not known, define: “Antibiotic stewardship” is an effort to limit unnecessary use of antibiotics, in order to prevent antibiotic resistance in the community and side effects in the patient.]
    a. How does this inform your clinical practice?
    b. Is there a culture of antibiotic stewardship at your clinical practice location? Please describe.
    c. What are the main challenges that clinicians like you face in implementing antibiotic stewardship?

VI. Utility and feasibility of eCDSTs

We are developing a clinical decision support tool that providers could use when a child with diarrhea presents to a clinic or hospital. Based on the patient’s clinical data and

population/environmental trends, the tool would provide evidence-based decision support for both *ordering diagnostic labs and also prescribing antibiotics.*

1. Electronic decision support tools can be used on a phone, tablet or computer. These tools usually ask a provider to input information about a patient, and then give you information that helps you make decisions about clinical care.
    a. Have you ever used an electronic clinical decision support tool to help you make a clinical decision? [If yes: Can you please share your experience with us about that?]
2. Would you be interested in using a tool to help you make decisions when caring for a child with diarrhea? (probe: why or why not)
3. [IF ANY DIAGNOSTIC TESTING AVAILABLE] Imagine a tool could give you guidance on whether or not to order a diagnostic test for a child with diarrhea. How do you think using a tool like this might change your practice on ordering clinical diagnostics, if at all?
4. Imagine a tool could give you guidance on whether or not to prescribe antibiotics for a child with diarrhea. How do you think using a tool like this might change your practice of using antibiotics, if at all?
5. What do you see as some of the challenges / drawbacks to using a clinical decision support tool when you treat children with diarrhea? (Probes: technical barriers, workload)
6. What would you like to see in a tool to help you manage diarrhea more efficiently?
    a. What advice do you have in designing a tool to help healthcare providers to manage pediatric diarrhea more efficiently?
    b. What would you like to see emphasized / included in this tool?
    c. How would you like to be trained in using this tool?

VII. Feedback on village doctors

1. Many families will first take their child with diarrhea to a village doctor. Do you have advice on how we can develop a tool to help village doctors make decisions about whether or not to prescribe antibiotics to children.

I have reached this end of my questions. Do you have anything to add on this topic?
